# TDP-43 pathology in anterior temporal pole cortex in aging and Alzheimer’s disease

**DOI:** 10.1186/s40478-018-0531-3

**Published:** 2018-05-01

**Authors:** Sukriti Nag, Lei Yu, Patricia A. Boyle, Sue E. Leurgans, David A. Bennett, Julie A. Schneider

**Affiliations:** 1 Rush Alzheimer Disease Center, Rush University Medical Center, Suite 1000, 1750 W Harrison Street, Chicago, IL 60612 USA; 2 Departments of Pathology (Neuropathology), Rush University Medical Center, Chicago, IL USA; 3 Departments of Neurological Sciences, Rush University Medical Center, Chicago, IL USA; 4 Departments of Behavioral Sciences, Rush University Medical Center, Chicago, IL USA

**Keywords:** Alzheimer’s disease, Anterior temporal pole, Dementia, Episodic memory, Hippocampal sclerosis, Orbital frontal cortex, Semantic memory, TDP-43

## Abstract

TDP-43 pathology was investigated in the anterior temporal pole cortex (ATPC) and orbital frontal cortex (OFC), regions often degenerated in frontotemporal lobar degenerations (FTLD), in aging and Alzheimer’s disease (AD). Diagnosis of dementia in the 1160 autopsied participants from 3 studies of community-dwelling elders was based on clinical evaluation and cognitive performance tests which were used to create summary measures of the five cognitive domains. Neuronal and glial TDP-43 cytoplasmic inclusions were quantitated in 8 brain regions by immunohistochemistry, and used in ANOVA and regression analyses. TDP-43 pathology was present in 547 (49.4%) participants in whom ATPC (41.9%) was the most frequently involved neocortical region and in 15.5% of these cases, ATPC was the only neocortical area with TDP-43 pathology suggesting not only that ATPC is involved early by TDP-43 but that ATPC may represent an intermediate stage between mesial temporal lobe involvement by TDP-43 and the last stage with involvement of other neocortical areas. To better study this intermediary neocortical stage, and to integrate with other staging schemes, our previous 3 stage distribution of TDP-43 pathology was revised to a 5 stage distribution scheme with stage 1 showing involvement of the amygdala only; stage 2 showed extension to hippocampus and/or entorhinal cortex; stage 3 showed extension to the ATPC; stage 4 – showed extension to the midtemporal cortex and/or OFC and finally in stage 5, there was extension to the midfrontal cortex. Clinically, cases in stages 2 to 5 had impaired episodic memory, however, stage 3 was distinct from stage 2 since stage 3 cases had significantly increased odds of dementia. The proportion of cases with hippocampal sclerosis increased progressively across the stages with stage 5 showing the largest proportion of hippocampal sclerosis cases. Stage 5 cases differed from other stages by having impairment of semantic memory and perceptual speed, in addition to episodic memory impairment. These data suggest that of the regions studied, TDP-43 pathology in the ATPC is an important early neocortical stage of TDP-43 progression in aging and AD while extension of TDP-43 pathology to the midfrontal cortex is a late stage associated with more severe and global cognitive impairment.

## Introduction

The transactive response DNA-binding protein 43 (TDP-43) was first localized in brains of cases with frontotemporal lobar degeneration (FTLD) and amyotrophic lateral sclerosis (ALS) [1, 20]. Subsequent studies localized this protein in Alzheimer’s disease (AD) [8, 24] and other neurodegenerative diseases such as age-related hippocampal sclerosis [14, 18], Lewy body (LB) diseases [11, 16] and chronic traumatic encephalopathy [12]. TDP-43 protein was also reported in the aging brain in the absence of a pathological diagnosis of AD [11, 15, 24] and in cognitively normal Asians [17]. The TDP-43 protein deposition was in the form of intranuclear or intracytoplasmic inclusions in neurons and glia, as well as dystrophic neurites in the affected regions; findings collectively referred to as TDP-43 pathology.

Few studies have investigated the regional distribution of TDP-43 pathology in the brain. In the behavioral variant FTLD, 4 patterns of TDP-43 distribution were reported [5] with early involvement of the orbital and inferior frontal gyri and anteromedial temporal structures to widespread cortical TDP-43 pathology, depending on the specific pattern. By contrast, TDP-43 distribution in AD was reported to follow 6 stages with medial temporal structures (stages 1-3) being affected early followed by ventral striatum, insular and inferior temporal cortices (stage 4), the brainstem (Stage 5) and finally basal ganglia and midfrontal cortex (stage 6) [8]. Our previous studies in a cohort of older community dwelling persons without FTLD, reported 3 stages of regional TDP-43 distribution regardless of presence or absence of AD [14, 15, 26]. In stage 1, TDP-43 was localized to the amygdala, in stage 2 there was extension of TDP-43 pathology to the hippocampus and/or entorhinal cortex while in stage 3 there was further extension to neocortical areas such as the midtemporal or midfrontal cortices.

TDP-43 pathology is considered central to the pathogenesis of FTLD/ALS whereas its role in other neurodegenerative diseases is less clear. Given the propensity for TDP-43 pathology to affect the anterior and/or orbital frontal cortex (OFC) and temporal lobe structures with prominent neurodegeneration in FTLD-TDP, the hypothesis that TDP-43 pathology may also preferentially involve the anterior temporal pole cortex (ATPC) and OFC in aging and AD was tested. ATPC is the most rostral neocortical area of the superior and middle temporal gyri and is highly interconnected with the amygdala and the OFC.

Our results show that the ATPC, but not the OFC, appears to be a prominent and early neocortical site of involvement in TDP-43 pathology associated with aging and AD and that this stage is related to dementia. To better study this early neocortical stage, and to integrate with other staging schemes, we propose a new 5 stage system of TDP-43 distribution that includes TDP-43 in ATPC. The association of all 5 stages of TDP-43 pathology with dementia, memory, and other cognitive domains was studied in participants of 3 longitudinal studies of aging and dementia: the Rush Memory and Aging Project (MAP), the Religious Orders Study (ROS) and the Minority Aging Research Project (MARS).

## Materials and methods

### Participants and clinical evaluation

Autopsied participants (*n* = 1160) were from 3 longitudinal clinical-pathologic cohort studies of aging and dementia, Rush MAP (*n* = 636), ROS (*n* = 501) and MARS (*n* = 23), each approved by the Institutional Review Board of Rush University Medical Center. All data collections (antemortem and postmortem) were similar in these studies allowing combined analyses of the cohorts. **A** signed, informed consent was obtained from each participant for an annual clinical evaluation and for brain donation. Fifteen cases having a pathologic diagnosis of FTLD in accordance with previous FTLD classifications [6, 10], were excluded from the study and are not included in the total number mentioned above. These excluded cases were diagnosed as having corticobasal degeneration, Pick’s disease, progressive supranuclear palsy, and FTLD-TDP. Thirty-four cases with missing tissue from any of the mandatory regions of interest were also excluded from the study. 18 cases having skipped areas (see below) were also excluded leaving 1108 cases available for statistical analyses. All 1108 cases had TDP-43 pathology data available from the amygdala since previous studies [8, 14] demonstrate that TDP-43 pathology in aging and AD appears to start and then spread from the amygdala.

Uniform clinical evaluation at baseline and annually thereafter included a standardized battery of 19 cognitive performance tests as described previously [3, 25]. The Mini-Mental State Examination (MMSE) and Complex Ideational Material were used for descriptive purposes or diagnostic classification, respectively. The remaining 17 tests assessed function of five cognitive domains including episodic, semantic, and working memory, perceptual speed and visuospatial ability. In order to reduce ceiling and floor artifacts as well as random variability, composite measures were obtained by converting the raw scores of the individual tests to z scores using the baseline mean and standard deviation (SD) of all participants and then averaging results for each domain of cognitive function [25].

Dementia and probable AD were diagnosed using criteria of the joint working group of the National Institute of Neurological and Communicative Disorders and Stroke and the AD and Related Disorders Association [13]. Dementia status proximate-to-death was assigned by a Board-certified neurologist after review of all clinical information.

### Pathological analyses

The average post-mortem interval was 9.3 h (SD 8.3). Brains were fixed with 4% paraformaldehyde in 0.1 M phosphate buffer. Blocks were dissected from 11 brain regions which included the following cortices: midfrontal (Brodmann9/46), midtemporal (Brodmann 21), inferior parietal (Brodmann 39/40), occipital (Brodmann17), anterior cingulate (Brodmann 24) and entorhinal (Brodmann 28) with amygdala. Blocks were also taken of the mid-hippocampus, basal ganglia at the level of the anterior commissure, anterior thalamus, midbrain at the level of the exiting 3rd nerve fibers and the cerebellum which included the dentate nucleus. Blocks were processed using standard techniques and paraffin-embedded sections (6 μm) stained with hematoxylin-eosin were used to detect microinfarcts and arteriolosclerosis as described below and hippocampal sclerosis (HS). The latter was evaluated unilaterally in a coronal section of the midhippocampus at the level of the lateral geniculate body, and graded as absent or present based on severe neuronal loss and gliosis in CA1 and/or subiculum or other sectors.

#### TDP-43 pathology

**Fig. 1:**
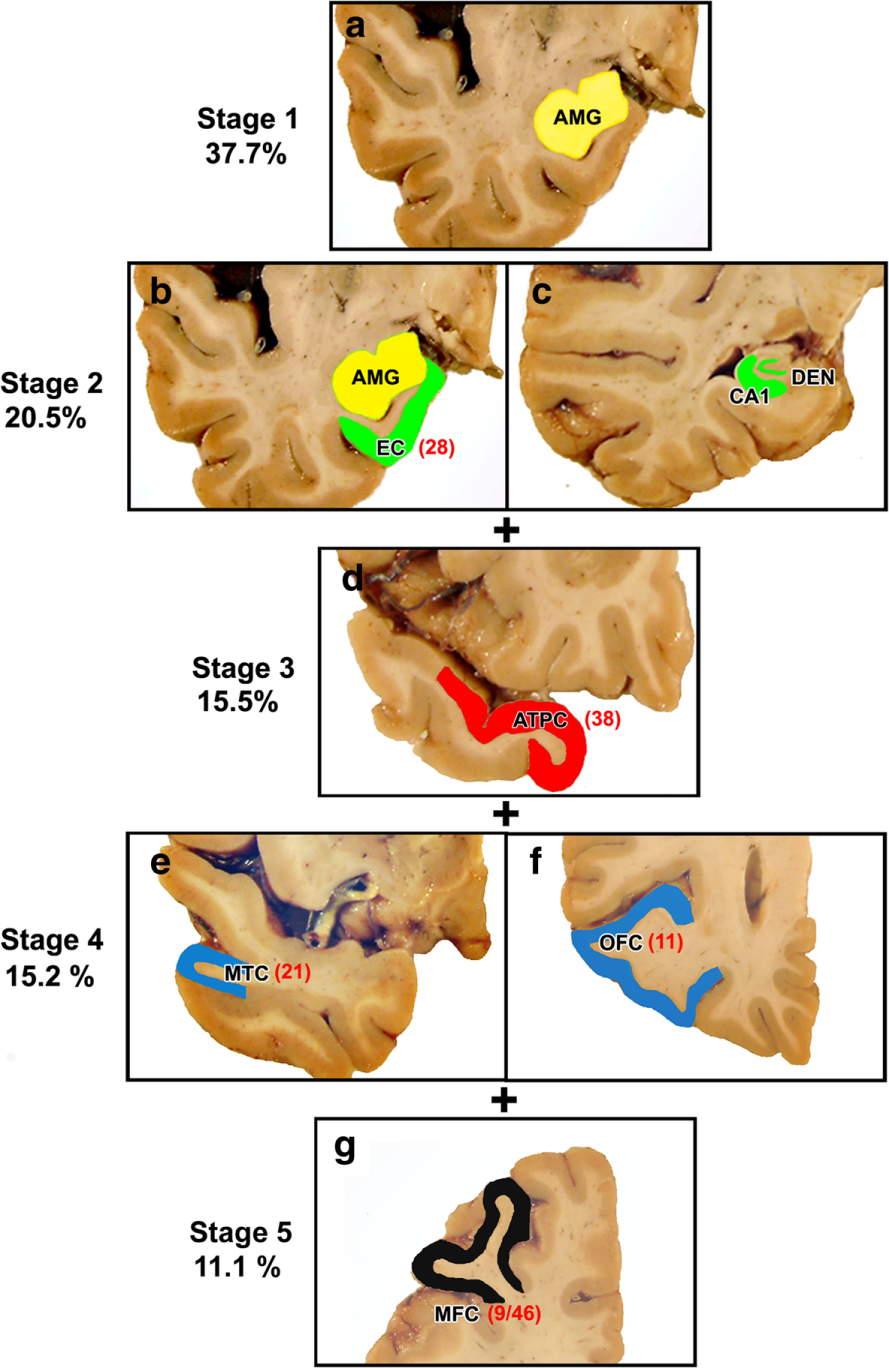
(**a**-**g**) The regional distribution of TDP-43 inclusions and percentage of cases showing TDP-43 inclusions in stages 1-5 are shown (**a**-**g**). This is a cumulative staging system such that any stage from 2 to 5 is considered to be positive if the previous stages are positive. The Brodmann designation of the cortices is shown in parentheses. AMG = amygdala, EC = entorhinal cortex, CA1 = CA1 sector of the hippocampus, DEN = dentate gyrus, ATPC = anterior temporal pole cortex, MTC = midtemporal cortex, OFC = orbital frontal cortex and MFC = midfrontal cortex

**Fig. 2:**
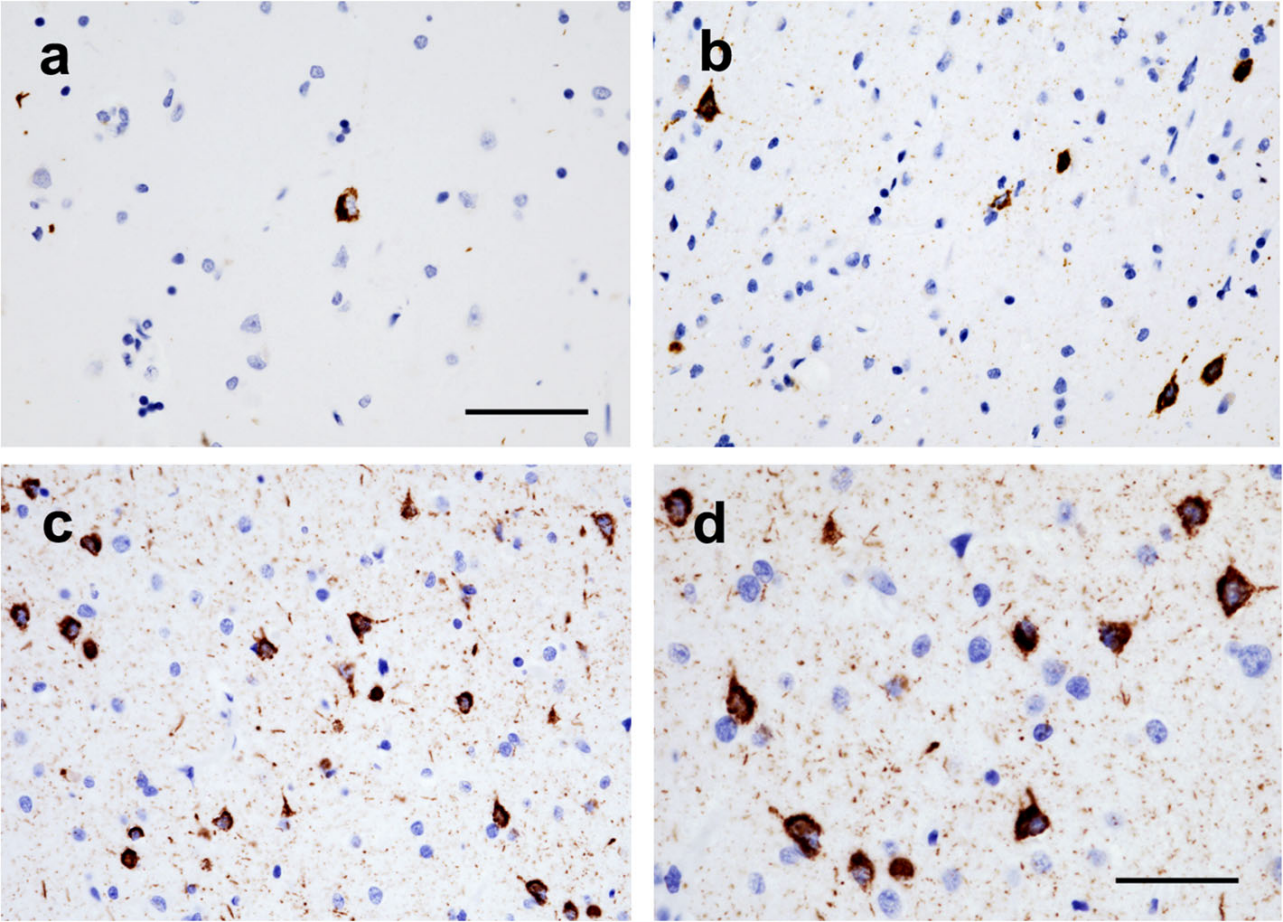
TDP-43 inclusions in neuronal cytoplasm and neurites in the ATPC are shown (a-d). Representative areas of the ATPC show sparse (**a**), moderate (**b**) and frequent (**c**) intracytoplasmic neuronal TDP-43 inclusions and neurite immunostaining. The areas depicted (**a**-**c**) are smaller than the 0.25 mm^2^ counting frame used to quantitate the inclusions. (**d**) Cytoplasmic TDP-43 in neurons and prominent neurite staining are shown in high magnification. Scale bar = 25 μm (**a**-**c**) and 50 μm (**d**)

TDP-43 protein was localized in four brain regions (amygdala, entorhinal cortex, hippocampus CA1 and subiculum and the dentate nucleus) and four neocortical areas (ATPC, midtemporal cortex, OFC and midfrontal cortex) having the Brodmann designation of 38, 21, 11 and 9/46 respectively (Fig. 1a-g). A phosphorylated monoclonal TAR5P-1D3 (pS409/410; 1:100, Ascenion, Munich, Germany) TDP-43 antibody [19] was used. A semiquantitative estimate of TDP-43 cytoplasmic inclusions in neurons and glia was obtained at 200 X, in a 0.25 mm^2^ area of greatest density using a 6-point scale (none, sparse [1-2 inclusions], sparse to moderate [3-5 inclusions], moderate [6-12 inclusions], moderate to frequent [13-19 inclusions], and frequent [20 or more inclusions]) (Fig. 2a-d). In analyses, a dichotomous variable was used to define presence of TDP-43 pathology in each region.

#### AD pathology

The National Institute on Aging-Reagan criteria [7] were used with intermediate and high likelihood cases indicating a pathologic diagnosis of AD. Modified Bielschowsky silver stain was used to quantitate neuritic and diffuse plaques and neurofibrillary tangles in 5 brain regions (midfrontal, midtemporal, inferior parietal and entorhinal cortices and hippocampus), having the highest density of these structures, as described previously [23]. The raw count for each of the three pathologies within each region was divided by the SD of each marker and values were averaged across the regions to obtain a summary score for each subject. The summary scores of these three AD markers were then averaged to yield the global measure of AD pathology for each subject, which was used in analyses.

#### Infarcts

The age, volume and anatomic location of all macroscopic infarcts and the age and location of microscopic infarcts were documented. Only chronic macro and microinfarcts were included in the analyses as dichotomous variables.

#### Vascular diseases

Atherosclerosis was assessed in basal cerebral arteries while arteriolosclerosis was assessed in the basal ganglia and both vessel pathologies were graded using a semiquantitative scale from 0 (none) to 6 (severe) as described previously [22]. Cerebral amyloid angiopathy (CAA) was assessed in meningeal and intracortical vessels in four cortical sections (midfrontal, midtemporal, inferior parietal and occipital) immunostained for β-amyloid and graded as described previously [27].

#### Lewy bodies

These were assessed in 6 regions (midfrontal, midtemporal, entorhinal and cingulate cortices, amygdala and substantia nigra) as described previously [25] and recorded and analyzed as a dichotomous variable.

Where pertinent, immunohistochemistry was done for anti-phospho-PHF tau pSer202/Thr205 (1:3000, Thermo Fisher Scientific, Waltham, MA) and ‘fused in sarcoma’ (FUS) protein (1:1000, Sigma Aldrich Corp, St. Louis, MO) to exclude FTLD cases. All immunohistochemistry was done using a Leica-Bond Max autostainer (Leica Microsystems, New Buffalo, IL). Antigen retrieval was done using heat-induced epitope retrieval (HIER) solution 1 (citrate-base) (Leica Microsystems) for 10 min for α-synuclein, and 30 min for FUS and HIER solution 2 (EDTA-base) (Leica Microsystems) for 20 min for phosphorylated TDP-43. Sections were pretreated with formic acid prior to immunostaining for β-amyloid. In case of α-synuclein the Bond polymer alkaline phosphatase red detection kit was used while for the other antibodies the Bond Polymer Refine Detection Kit was used which produced a brown reaction product.

### Statistical analyses

Demographics, clinical characteristics and age-related pathologies including macro and microinfarcts, HS, LBs and AD pathologies and vascular pathologies (arteriolosclerosis, atherosclerosis, and CAA), were compared between subjects without and those in the five TDP-43 stages using χ^2^ or the analysis of variance (ANOVA). Age, education, MMSE and cognitive domains were further compared by post hoc pairwise comparisons between TDP-43 stages 2 and 3, stages 3 and 4 and stages 4 and 5 cases with application of a Bonferroni Correction (α = 0.05/3) to adjust for multiple testing.

Multivariable linear regression analyses were used to determine the association of the 5 TDP-43 pathology stages with the outcome measures of episodic, semantic, and working memory, perceptual speed and visuospatial skills. Multivariable logistic regression analyses evaluated the association of the five stages of TDP-43 pathology with dementia as a binary outcome. In both multivariable linear and logistic regression analyses, Stage 0 (no TDP-43 pathology) was used as the reference group and all models controlled for age, sex, education and the age-related pathologies listed above. All analyses were carried out using SAS software, (SAS Institute Inc. SAS/STAT 14.1 User’s Guide, Cary, NC). Model assumptions were examined graphically and analytically and were adequately met. A nominal threshold of *p* < 0.05 was used for statistical significance throughout except for the post hoc pairwise comparisons mentioned above.

## Results

TDP-43 neuronal and glial inclusions were present in 547 of 1108 (49.4%) participants. TDP-43 cytoplasmic inclusions in entorhinal and neocortical regions tended to be more frequent in the second layer than in deeper cortical layers. Most inclusions were compact (Fig. 2d) while granular inclusions were less frequent.

### TDP-43 in ATPC and OFC

In the TDP-43 positive cases, the most frequent neocortical area showing TDP-43 inclusions was the ATPC (41.9%) followed by the midtemporal cortex, the OFC and inclusions were least common in the midfrontal cortex (Table 1). In 15.5% of the 547 cases, ATPC was the only neocortical area showing TDP-43 pathology suggesting that ATPC represents one of the earliest sites of neocortical involvement in the progression of TDP-43 pathology and that it may represent an intermediate stage between mesial temporal lobe involvement by TDP-43 pathology and the last stage with more extensive neocortical involvement. Extension of TDP-43 pathology to the midfrontal cortex (see below), had a distinct pathological and clinical profile justifying separation of these cases into an additional stage. We therefore revised our 3 stage distribution of TDP-43 pathology to include two additional stages. Our new 5 stage distribution of TDP-43 pathology was as follows: stage 1 – localized to amygdala; stage 2 –extension to hippocampus and/or entorhinal cortex; stage 3 – extension to the ATPC, stage 4 – extension to other neocortical areas such as midtemporal or OFC and finally in stage 5 there was extension to the midfrontal cortex (Fig. 1a-g). Stages 2 and 4 were considered to be positive if any of the new regions included in these stages showed TDP-43 pathology.

**Table 1:** Frequency of TDP-43 pathology in brain regions by stage in 547 participants

Regions	TDP-43 stages					Total TDP-43 positive cases by brain regions n, %
	1	2	3	4	5	
	*n = 206*	*n = 112*	*n = 85*	*n = 83*	*n = 61*	
	37.7%	20.5%	15.5%	15.2%	11.1%	
Amygdala	206	112	85	83	61	547, *100*
Entorhinal Cortex	0	92	83	82	60	317, *58.0*
Hippocampus, CA1	0	84	74	82	59	299, *54.7*
Hippocampus, dentate gyrus	0	60	59	76	59	244, *44.6*
Anterior temporal pole cortex	0	0	85	83	61	229, *41.9*
Midtemporal cortex	0	0	0	70	57	127, *23.2*
Orbital frontal cortex	0	0	0	32	57	89, *16.3*
Midfrontal cortex	0	0	0	0	61	61, *11.1*

**Fig. 3:**
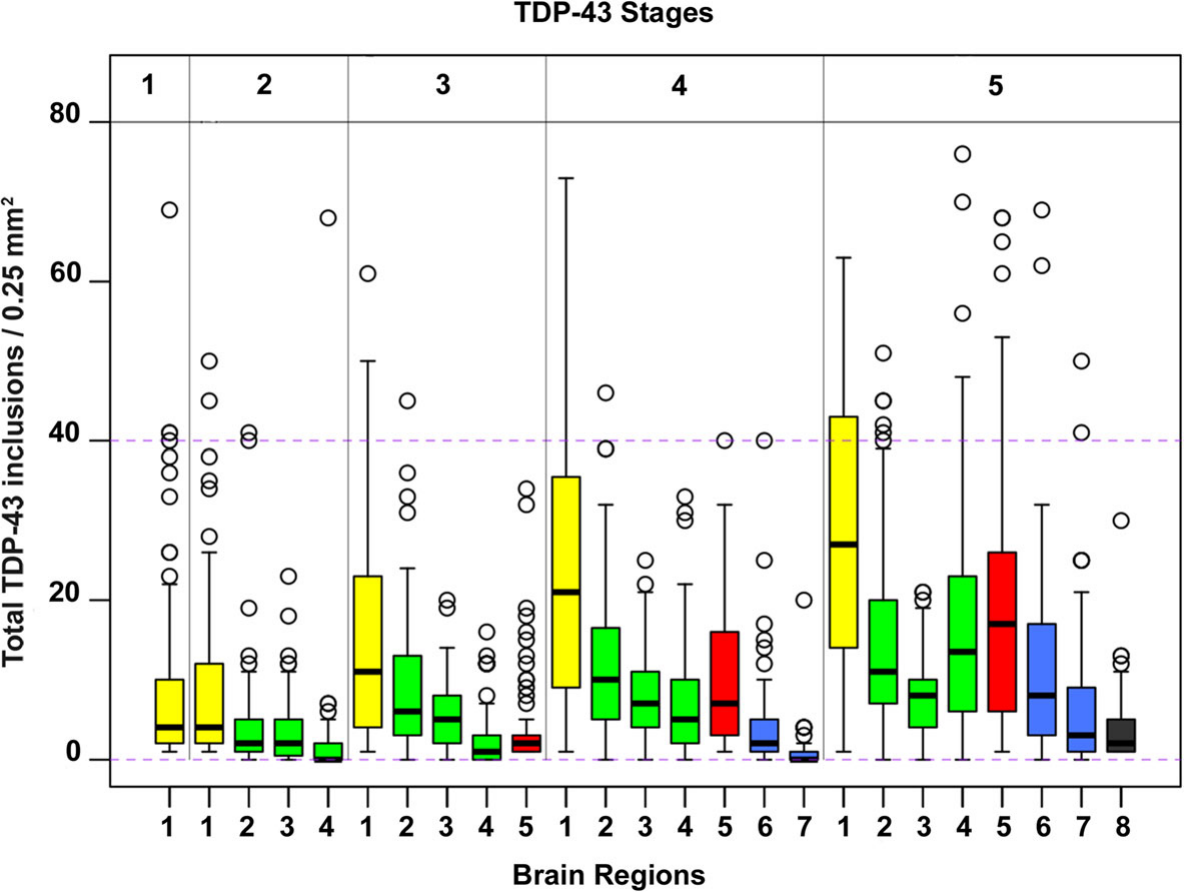
Box plots showing the total number of TDP-43 inclusions per 0.25 mm^2^ area in the eight brain regions by stage. The numbers on the x axis denote the brain regions which are designated as 1: amygdala, 2: entorhinal cortex, 3:CA1 sector of the hippocampus, 4: dentate neurons of the hippocampus, 5: anterior temporal pole cortex, 6: midtemporal cortex, 7: orbital frontal cortex, 8: midfrontal cortex. There is progressive increase of inclusions in the amygdala by stage. Inclusions in all regions including the ATPC are maximal in stage 5

All participants having TDP-43 pathology (*n* = 547), showed inclusions in the amygdala and in 206 of the 547 cases (37.7%), the inclusions were confined to the amygdala (stage 1) (Fig. 1a, Table 1). Extension of TDP-43 to the entorhinal cortex or CA1 sector of the hippocampus or dentate neurons was observed in 112 of the 547 (20.5%) cases (stage 2). Further extension of TDP-43 to the ATPC (stage 3) was observed in 85 of the 547 (15.5%) cases while additional extension to midtemporal or OFC (stage 4) was observed in 83 of the 547 (15.2%) cases and extension to the midfrontal cortex (stage 5) was observed in 61 of the 547 (11.1%) cases. In both stage 4 and 5 cases the number of TDP-43 inclusions in ATPC was greater than observed in stage 3 cases (Fig. 3). Stage 4 or 5 cases did not show significant microvacuolation or obvious neurodegeneration of the frontal and/or midtemporal cortices that characterize FTLD cases. Nine cases in stage 5, having no dementia (Table 2) did not have AD but other pathologies such as HS (*n* = 4), a combination of HS and LB disease (n = 4) and 1 case had a chronic macroinfarct in the caudate. Phospho-PHF-tau and FUS immunostaining done in these cases was negative excluding a diagnosis of FTLD.

**Table 2:** Clinical pathologic characteristics of 1108 participants by TDP-43 stages

Characteristics	TDP-43 Stages						*p*-value
	Stage 0 *n = 561*	Stage 1 *n = 206*	Stage 2 *n = 112*	Stage 3 *n = 85*	Stage 4 *n = 83*	Stage 5 *n = 61*	
Age at death, y, mean (SD)	87.7 (6.9)	89.6 (6.6)	91.5 (6.1)	92.0 (5.6)	92.1 (5.3)	90.3 (5.3)	< 0.001*
Female, n (%)	370 (66.0)	144 (69.9)	79 (70.5)	67 (78.8)	67 (80.7)	41 (67.2)	0.036
Education, mean (SD),	16.1 (3.9)	16.2 (3.6)	16.1 (3.6)	15.7 (3.5)	15.5 (3.4)	15.8 (3.3)	0.705*
*Clinical characteristics, n (%)*							
No Dementia	376 (67.4)	130 (63.1)	55 (49.1)	30 (35.3)	27 (32.9)	9 (14.8)	< 0.001
Dementia	182 (32.6)	76 (36.9)	57 (50.9)	55 (64.7)	55 (67.1)	52 (85.3)	
*Cognitive function tests proximate to death, mean (SD)*							
MMSE score	22.8 (8.2)	21.5 (8.6)	19.0 (9.8)	18.3 (9.5)	15.6 (9.2)	11.2 (10.5)	< 0.001*
Episodic memory	−0.60 (1.3)	−0.71 (1.3)	−1.23 (1.4)	−1.38 (1.3)	−1.97 (1.2)	−2.19 (1.3)	< 0.001*
Semantic memory	−0.91 (1.5)	−1.05 (1.5)	−1.63 (1.9)	−1.51 (1.7)	−1.96 (1.7)	−2.80 (2.1)	< 0.001*
Working memory	−0.61 (1.1)	−0.65 (1.1)	− 0.95 (1.2)	−0.87 (1.0)	− 0.97 (1.0)	− 1.27 (1.3)	< 0.001*
Perceptual speed	− 1.03 (1.2)	− 1.08 (1.1)	− 1.42 (1.2)	−1.51 (1.1)	− 1.68 (1.1)	−2.10 (1.2)	< 0.001*
Visuospatial ability	− 0.42 (1.1)	−0.41 (1.2)	− 0.64 (1.2)	−0.68 (1.1)	− 0.91 (1.1)	− 1.05 (1.3)	< 0.001*
*Pathologic characteristics, n (%)*							
AD, NIA-Reagan	315 (56.2)	136 (66.0)	75 (67.0)	68 (80.0)	71 (85.5)	52 (85.3)	< 0.001
Hippocampal sclerosis	10 (1.8)	7 (3.4)	14 (12.5)	14 (16.5)	27 (32.5)	38 (62.3)	< 0.001
Macroinfarcts	196 (34.9)	77 (37.4)	38 (33.9)	29 (34.1)	37 (44.6)	21 (34.4)	0.724
Microinfarcts	158 (28.2)	61 (29.6)	36 (32.1)	21 (24.8)	26 (31.3)	23 (37.7)	0.678
Arteriolosclerosis	161 (28.9)	76 (37.1)	32 (28.6)	33 (38.8)	30 (36.1)	20 (29.5)	0.145
Atherosclerosis	174 (31.1)	71 (34.5)	37 (33.0)	27 (31.8)	28 (33.7)	20 (32.8)	0.966
Cerebral amyloid angiopathy	418 (75.2)	155 (75.6)	95 (84.8)	69 (81.2)	66 (79.5)	51 (83.6)	0.171
Lewy body disease	115 (21.1)	50 (24.9)	32 (29.4)	24 (29.6)	16 (20.5)	25 (40.0)	0.008

*p*-value derived from ANOVA* or chi-square

18 of the 1160 (1.5%) cases failed to show the proposed regional progression of TDP-43 pathology due to one skipped stage despite examination of an additional section of the regions without identified TDP-43 pathology. Of the 18 cases, eight showed no TDP-43 pathology in the amygdala which showed no degenerative changes on microscopy. In 5 cases the hippocampus/entorhinal cortex was skipped, in another 3 cases the ATPC was skipped and in 2 cases the midtemporal cortex was skipped. The clinical diagnoses of these 18 cases were probable AD (6 cases), mild cognitive impairment (7 cases) and no cognitive impairment (5 cases). Fourteen of the 18 cases met criteria for pathologic AD while the remaining 4 cases did not, however, numerous macroinfarcts/microinfarcts with significant tissue loss was present in these cases. These18 cases were not included in the statistical analyses.

### TDP-43 stages and age-related pathologies

Overall, the frequency of a pathologic diagnosis of AD was higher in those having TDP-43 pathology (73.5%) as compared to those negative for TDP-43 (56.2%). The proportion of cases with a pathologic diagnosis of AD increased across the TDP-43 stages to 80% in Stage 3 and 85% in each of stages 4 or 5 (Table 2). Bivariate analyses showed that HS frequency was 10-fold higher in cases having TDP-43 pathology as compared to those without TDP-43 pathology. In addition, a progressive increase in percentage of HS was noted across the TDP-43 stages with a nine-fold increase in HS frequency in stage 3 cases and a 35-fold increase in stage 5 cases. Lewy body disease was significantly higher in those with TDP-43 pathology as compared to those having no TDP-43 with a 2-fold increase in frequency in the stage 5 cases as compared with those without TDP-43 pathology. The frequencies of other age-related pathologies including macro and microinfarcts, and vessel pathologies such as arteriolosclerosis, atherosclerosis, and CAA did not differ by TDP-43 pathology.

### Clinical findings in TDP-43 stages

The demographic and clinical data for participants in each of the 5 TDP-43 stages are shown in Table 2. Overall, age was significantly higher in cases with TDP-43 pathology as compared to those without TDP-43 pathology (*p* < 0.001). Post hoc pairwise comparisons between the age of stage 2 and 3 cases, stage 3 and 4 cases and stage 4 and 5 cases showed no statistical difference (*p* = 0.574, *p* = 0.945 and *p* = 0.104 respectively). Frequency of females was slightly higher in those having TDP-43 pathology while education did not differ by TDP-43 status. Of the cases having no TDP-43 pathology, 67% had no dementia and the percentage of participants with no dementia decreased across the TDP-43 stages with only 15% showing no dementia in stage 5 (Table 2).

### Relation of TDP-43 stages to dementia

The mean MMSE score, proximate to death was 22.8 (SD 8.2) in the group without TDP-43 pathology while the mean MMSE score was significantly lower in those having TDP-43 pathology being 18.5 (SD 9.8). The MMSE scores were progressively lower across TDP-43 stages 2 to 5 (Table 2). Post hoc pairwise comparisons between the mean MMSE scores of stages 2 and 3 and stages 3 and 4 cases showed no difference while comparison between stages 4 and 5 cases showed significantly lower (*p* = 0.003) MMSE scores in the stage 5 cases. Of the cases without TDP-43 pathology, one-third had dementia. The percentage frequency of dementia increased across the TDP-43 stages with 65% of stage 3, and 85% of stage 5 cases having dementia.

In logistic regression analyses, controlling for demographics and other age-related pathologies, higher odds of dementia were observed in TDP-43 stages 3 (odds ratio 2.68, confidence interval 1.51-4.75, *p* < 0.001), 4 (odds ratio 1.90, confidence interval 1.05-3.42, *p =* 0.034) and 5 cases (odds ratio 5.20, confidence interval 2.23-12.1, *p* < 0.001) as compared to those without TDP-43 pathology (Table 3). Additional models with dementia as an outcome included interaction terms between the TDP-43 stages and AD, or LB disease or HS. These interaction terms were not significant suggesting that the association of the TDP-43 stages with dementia were not affected by the presence of these diseases.

**Table 3:** Relation of the five TDP-43 positive stages and age-related pathologies to dementia

Model terms	Relation to dementia	
	OR (95% CI)	*p*-value
TDP-43 stage 1^a^	0.91 (0.62,1.35)	0.645
TDP-43 stage 2^a^	1.38 (0.84, 2.24)	0.203
TDP-43 stage 3^a^	2.68 (1.51, 4.75)	< 0.001
TDP-43 stage 4^a^	1.90 (1.05, 3.42)	0.034
TDP-43 stage 5^a^	5.20 (2.23, 12.1)	< 0.001
AD Pathology	3.81 (2.93, 4.96)	< 0.001
Macroinfarcts	2.27 (1.66, 3.11)	< 0.001
Microinfarcts	1.26 (0.91,1.74)	0.167
Atherosclerosis	1.48 (1.07, 2.04)	0.019
Arteriolosclerosis	1.38 (1.00,1.90)	0.054
Lewy bodies	3.09 (2.20, 4.34)	< 0.001
Hippocampal sclerosis	3.48 (1.86, 6.49)	< 0.001

Estimated from multiple logistic regression models, adjusted for age at death, sex and education

^a^represents contrasts with TDP-43 stage 0

### TDP-43 stages and cognitive domains

Overall impairment of cognitive domains was greater in those with TDP-43 pathology as compared with those without TDP-43 pathology (*p* < 0.001). In those with TDP-43 pathology impairment of specific cognitive domains was varied by the TDP-43 stage (Table 2). The mean scores for the cognitive domains of episodic, semantic, working memory, perceptual speed and visuospatial ability were progressively lower across TDP-43 stages 2 through 5 with lowest values in stage 5 cases. Post hoc pairwise comparisons between stages 2 and 3 cases showed no difference in the values of the five cognitive domains. Comparison of stage 3 and 4 cases showed that episodic memory was more impaired (*p =* 0.003) in the stage 4 cases while impairment of semantic memory was greater (*p* = 0.002) in stage 5 cases as compared to stage 4 cases.

Using linear regression models which controlled for demographics, degenerative and vascular pathologies and infarcts, cases with TDP-43 stages 2 through 5 had a lower level of episodic memory as compared to those without TDP-43 pathology (Table 4). Only Stage 5 subjects showed additional impairment in semantic memory, and perceptual speed.

**Table 4:** Relation of TDP-43 stages and age-related pathologies to cognitive outcomes

Model terms	Estimate (SE) *p-v*alue				
	Episodic Memory	Semantic Memory	Working Memory	Perceptual Speed	Visuospatial Abilities
TDP-43 stage 1^a^	0.05 (0.09) 0.591	0.06 (0.12) 0.592	0.08 (0.09) 0.374	0.10 (0.09) 0.273	0.12 (0.09) 0.174
TDP-43 stage 2^a^	−0.32 (0.12) 0.008	− 0.34 (0.15) 0.024	− 0.16 (0.11) 0.141	− 0..12 (0.12) 0.290	− 0.02 (0.11) 0.874
TDP-43 stage 3^a^	− 0.31 (0.14) 0.023	− 0.06 (0.17) 0.717	0.02 (0.13) 0.879	− 0.13 (0.13) 0.337	0.07 (0.13) 0.594
TDP-43 stage 4^a^	− 0.76 (0.15) < 0.001	− 0.34 (0.18) 0.064	−0.02 (0.13) 0.886	− 0.22 (0.14) 0.108	−0.08 (0.14) 0.574
TDP-43 stage 5^a^	−0.83 (0.18) < 0.001	− 0.97 (0.22) < 0.001	−0.24 (0.16) 0.142	− 0.61 (0.17) < 0.001	−0.20 (0.17) 0.254
AD pathology	−0.90 (0.06) < 0.001	− 0.96 (0.07) < 0.001	−0.55 (0.05) < 0.001	−0.54 (0.06) < 0.001	−0.40 (0.06) < 0.001
Macroinfarcts	−0.40 (0.08) < 0.001	−0.42 (0.10) < 0.001	−0.37 (0.07) < 0.001	−0.25 (0.07) < 0.001	−0.12 (0.07) 0.097
Microinfarcts	−0.08 (0.08) 0.288	−0.15 (0.10) 0.134	− 0.01 (0.07) 0.845	−0.14 (0.07) 0.053	− 0.01 (0.07) 0.905
Atherosclerosis	−0.14 (0.08) 0.078	− 0.10 (0.10) 0.321	−0.05 (0.07) 0.465	− 0.15 (0.07) 0.041	−0.14 (0.07) 0.063
Arteriolosclerosis	−0.23 (0.08) 0.003	−0.20 (0.10) 0.039	− 0.13 (0.07) 0.063	−0.19 (0.07) 0.011	− 0.12 (0.07) 0.107
Lewy body disease	−0.44 (0.08) < 0.001	− 0.79 (0.10) < 0.001	−0.42 (0.07) < 0.001	− 0.42 (0.08) < 0.001	−0.28 (0.08) < 0.001
Hippocampal sclerosis	−0.59 (0.14) < 0.001	− 0.66 (0.17) < 0.001	− 0.24 (0.12) 0.055	−0.21 (0.13) 0.100	− 0.34 (0.13) 0.009

Cognitive outcomes are estimated from separate linear regressions, all adjusted for age at death, sex, and education. Cell entries are estimate, standard error (SE), and probability value

^a^represent contrasts with TDP-43 stage 0

## Discussion

This clinical-pathologic study of community-dwelling older subjects focuses on the pathological and clinical significance of detecting TDP-43 pathology in the ATPC, OFC and midfrontal cortex. Our results show that TDP-43 pathology in the ATPC, but not in the OFC, represents an early neocortical stage in the progression of TDP-43 pathology in aging and AD being intermediate between stage 2 (mesial temporal) and the later stages with more widespread neocortical TDP-43 distribution and that involvement of the ATPC is an important pathologic marker of the transition to dementia. While episodic memory is impaired in stages 2-5, significantly lower MMSE scores, impairment of semantic memory and perceptual speed only occurs when TDP-43 protein involves the midfrontal cortex.

Although the ATPC is known to be degenerated in FTLD [2, 9], and AD [2], there are no reports of TDP-43 protein localization in this region. Several studies of AD [8, 11] and elders without a pathological diagnosis of AD [15, 17] have documented the regional distribution of TDP-43 pathology which occurs in a stereotyped manner with involvement of amygdala and medial temporal structures before involvement of the neocortex. In the present study, the ATPC was the most frequently involved neocortical area while OFC involvement was less frequent, although somewhat greater than the midfrontal cortex. Since the ATPC is the most frequently involved neocortical area showing TDP-43 pathology, adding this region to TDP-43 staging protocols will detect more cases having a higher TDP-43 stage with early neocortical involvement. The late involvement of the OFC (stage 4), differentiates the present cases from the FTLD cases which show early involvement of this region [5]. Overall, it appears that TDP-43 pathology in aging is a temporal lobe predominant neurodegenerative disorder with early involvement of the mesial and anterior temporal lobe structures and later involvement of frontal cortex. In 11% of cases there was extension of TDP-43 pathology to the midfrontal cortex which represents the last stage of TDP-43 distribution. In this respect the present staging is similar to the 6 stage TDP-43 distribution scheme in AD in which midfrontal cortex was involved last [8]. However, the present TDP-43 staging is simplified since the last stage was reached by sectioning 8 regions instead of the 14 regions studied in the previous paper and with the present scheme, cases can be staged if for example the brainstem or subcortical regions are unavailable.

Eighteen of the 1160 cases (1.5%) failed to show the proposed regional progression of TDP-43 pathology due to one skipped stage. Since additional sections of these skipped regions were examined and significant neuronal loss was not detected, lack of TDP-43 pathology in these regions was ascribed to the biological variation of TDP-43 distribution. Skipped regions were also reported in other studies of TDP-43 staging [8, 17].

TDP-43 pathology in the ATPC was associated with a higher percentage of cases with pathologic AD and slightly higher percentage of cases with HS pathology compared to TDP-43 involvement limited to the hippocampus or entorhinal cortex (stage 2). These observations support the ATPC being an intermediate pathologic stage of age-related TDP-43 proteinopathy. The observation that the frequency of AD was significantly higher in cases as the TDP-43 stage increases beyond the amygdala was reported previously [11, 14]. Also, the frequency of HS in cases having TDP-43 pathology was 10 fold that observed in those without TDP-43 pathology. In addition, the percentage of HS was higher in the higher TDP-43 stages. Increased frequency of TDP-43 pathology in cases with HS [11, 14, 26] and increased frequency of HS in the higher TDP-43 stages [8, 14] has been noted previously. Frequencies of macro and microinfarcts and vascular diseases were not different in those without and those with TDP-43 pathology including those with involvement of the ATPC. As reported previously [11, 14, 15, 24], there was coexistence of TDP-43 pathology and LB disease, with a significant increase in LB disease in those having TDP-43 pathology as compared to those without TDP-43 pathology. The interrelationship between the TDP-43 pathology stages and these co-morbid pathologies require further study.

TDP-43 pathology stages 3 through 5 were associated with higher odds of dementia which was independent of coexisting pathologic diagnoses of AD, LB disease or HS, suggesting that the progression of TDP-43 from the mesial temporal lobe to the ATPC represents an important transition and likely marks the onset of more severe functional changes.

Impaired episodic memory, a significant finding in AD was present across all the TDP-43 stages except stage 1. The independent association of TDP-43 pathology with impaired episodic memory was also observed in our previous studies of mixed AD and non-AD cases [14, 26] and in community dwelling elders without a pathologic diagnosis of AD or FTLD [15]. Post hoc pairwise comparisons emphasized the differences in the cognitive profiles of stage 3, 4 and 5 cases with impairment of semantic memory occurring only in stage 5 cases. Linear regression did not show an association between stage 3 and 4 cases with semantic memory. Possibly impairment of this modality only occurs with more widespread TDP-43 pathology localization in the midfrontal cortex suggesting that degenerative/functional changes occur as the pathology accumulates. In addition, although ATPC and the midtemporal cortex are known to have a critical role in semantic representation and/or processing [21], the neuroanatomic model of semantic processing is more extensive than just temporal cortex with seven regions (angular, middle temporal, fusiform and parahippocampal, inferior frontal and posterior cingulate gyri, dorsomedial and ventromedial prefrontal cortices) being consistently engaged during functional magnetic resonance imaging and positron emission tomography of subjects [4].

Strengths of this study include detailed data on multiple neuropathologies on a large number of participants obtained in a blinded manner and the availability of detailed clinical data on diagnosis and neuropsychological testing of these participants performed proximate to death. These studies also have high follow-up and autopsy rates that provide internal validity of findings.

A perceived limitation could be the lack of TDP-43 data from the basal ganglia and brainstem. Although, the staging presented in this study is contracted as compared to a previous study of TDP-43 staging in AD [8], the highest stage in both studies shows involvement of the midfrontal cortex. Another potential limitation may be that only one hemisphere was sampled raising the possibility of misclassification. While over half the participants were derived from the community, many were from ROS and these participants likely had better dietary intake, access to health care and levels of education, all factors affecting cognitive risk. The number of minorities in this study is small therefore further studies will be required of minority cohorts.

## Conclusions

Pathological and clinical data justified expansion of our previous 3 stage TDP-43 pathology distribution scheme to a 5 stage distribution scheme. Of the neocortical areas studied, ATPC was the earliest neocortical region showing TDP-43 pathology therefore TDP-43 pathology involves the temporal cortex earlier than the frontal cortex. Cases with TDP-43 in the ATPC had significantly increased odds of dementia justifying separation of ATPC to a distinct stage. Impairment of episodic memory was present in stages 2 to 5 but impairment of semantic memory and perceptual speed was only observed when TDP-43 pathology spread from the temporal cortex to involve the midfrontal cortex.

## Abbreviations

AD: Alzheimer’s disease
ALS: Amyotrophic lateral sclerosis
ANOVA: Analysis of variance
ATPC: Anterior temporal pole cortex
CAA: Cerebral amyloid angiopathy
FTLD: Fronto-temporal lobar degeneration
HS: Hippocampal sclerosis
LB: Lewy body
MAP: Memory and Aging Project
MARS: Minority Aging Research Project
MMSE: Mini-mental state examination
OFC: Orbital frontal cortex
ROS: Religious Orders Study
SD: Standard deviation
TDP-43: Transactive response DNA-binding protein 43
